# Exploring the Role of Laryngopharyngeal Reflux in Eustachian Tube Dysfunction: Therapeutic Potential of Proton Pump Inhibitors in a Resource-Constrained Setting

**DOI:** 10.7759/cureus.83896

**Published:** 2025-05-11

**Authors:** Muhammad Tehseen Ahmad, Umer Farooq, Arslan Akram, Syeda Hadia Batool Naqvi, Maimoona Maheen, Ali Haider, Abdulqadir J Nashwan, Shahzaib Maqbool, Abdur Rehman

**Affiliations:** 1 Otolaryngology, Benazir Bhutto Hospital, Rawalpindi, PAK; 2 Cardiology, Rawalpindi Medical University, Sialkot, PAK; 3 Internal Medicine, Rawalpindi Medical University, Rawalpindi, PAK; 4 Internal Medicine, Nishtar Medical University, Multan, PAK; 5 Medicine, Rawalpindi Medical University, Rawalpindi, PAK; 6 Internal Medicine, Hamdard College of Medicine and Dentistry, Karachi, PAK; 7 Nursing and Midwifery, Hamad Medical Corporation, Doha, QAT; 8 Pediatric Surgery, Rawalpindi Medical University, Rawalpindi, PAK; 9 Otolaryngology, Rawalpindi Medical University, Rawalpindi, PAK

**Keywords:** asthma, chronic cough, eustachian tube dysfunction, eustachian tube dysfunction questionnaire, extraesophageal manifestations, gastroesophageal reflux disease, laryngitis, laryngopharyngeal reflux disease, proton pump inhibitors (ppis), quality-of-life

## Abstract

Background

Eustachian tube dysfunction (ETD) and laryngopharyngeal reflux disease (LPRD) are under-recognized conditions that significantly impact quality of life. LPRD may contribute to ETD via gastric refluxate-induced mucosal damage. While proton pump inhibitors (PPIs) are commonly used for reflux management, their role in alleviating ETD symptoms remains controversial. This study investigates the association between LPRD and ETD and evaluates the efficacy of PPI therapy in ETD management.

Methods

A prospective study was conducted on patients diagnosed with ETD and LPRD. A total of 126 patients were included using consecutive non-randomized sampling. The participants underwent an eight-week regimen of twice-daily 40 mg oral omeprazole. ETD severity was assessed using the Eustachian Tube Dysfunction Questionnaire (ETDQ-7), while LPRD severity was measured via the Reflux Symptom Index (RSI) and Reflux Finding Score (RFS), both pre- and post-intervention. Independent t-tests and correlation analyses were performed to evaluate treatment outcomes.

Results

Significant reductions were observed in ETDQ-7 (16.20±10.47 to 10.40±5.47, p<0.001), RSI (11.42±7.65 to 4.50±2.65, p<0.001), and RFS (22.56±10.14 to 4.84±2.45, p<0.001) scores following PPI therapy, suggesting symptomatic improvement in both ETD and LPRD. Correlation analysis revealed a moderate association between both ETDQ-7 and RSI (0.346-0.391, p<0.001), and ETDQ-7 and RFS (0.437-0.515, p<0.001) scores respectively. However, residual symptoms in some patients suggest that additional pathophysiological factors may contribute to ETD beyond the reflux-mediated inflammation.

Conclusion

This study provides evidence supporting the link between LPRD and ETD and suggests that PPI therapy may alleviate ETD symptoms in a subset of patients. However, given the multifactorial nature of ETD, further studies are needed to explore alternative therapies (such as potassium channel blockers, Eustachian tube dilation, etc.) and long-term treatment outcomes.

## Introduction

Eustachian tube dysfunction (ETD) is a clinical condition marked by the inability of the Eustachian tube to adequately ventilate the middle ear, resulting in symptoms like aural fullness, hearing difficulties, and middle ear pressure abnormalities [1]. It is an under-recognized condition with significant implications for the quality of life of patients, including social and psychological impact [2]. It is diagnosed by a combination of patient-reported symptoms, nasal and otoscopic examination, and specialized tests that include, but are not limited to, tympanometry and audiometry [1]. Similarly, laryngopharyngeal reflux disease (LPRD) represents a complex, extra-esophageal manifestation of gastroesophageal reflux disease (GERD), which causes chronic irritation and inflammation of the upper aerodigestive tract [3]. Emerging evidence has pointed to a potential pathogenic interaction between these two conditions, emphasizing their shared anatomical proximity and inflammatory pathways [4].

The pathophysiological link between ETD and LPRD seems to lie in the exposure of the nasopharyngeal region to gastric refluxate containing acid and pepsin. This exposure is believed to impair the mucosal barrier of the Eustachian tube, disrupt its pressure regulation mechanisms, and ultimately result in dysfunction [5]. Recent evidence suggests that LPRD contributes to ETD through direct mucosal irritation and inflammation-induced edema, impairing the opening of the tube and middle ear ventilation [6]. Acidic refluxate has been detected in the middle ear effusions of patients with chronic otitis media, further supporting a potential role for LPRD in the pathophysiology of ETD [7]. However, while a growing body of literature supports this hypothesis, the clinical implications remain poorly defined, particularly in resource-constrained settings like Pakistan. Here, the dual burden of untreated reflux disorders and otologic conditions is exacerbated by limited access to healthcare and diagnostic resources.

Although tools like the Eustachian Tube Dysfunction Questionnaire (ETDQ-7) and the Reflux Symptom Index (RSI) have been validated for diagnosing these conditions in controlled environments, their real-world applicability in low-resource settings is rarely studied [8,9]. Moreover, therapeutic interventions, particularly the role of proton pump inhibitors (PPIs), remain controversial due to a lack of evidence to formulate a proper treatment regimen. While PPIs effectively reduce laryngopharyngeal inflammation by suppressing gastric acid secretion, their impact on ETD symptoms has been less extensively studied [4,10]. A prospective interventional study by Park et al. reported symptomatic relief in patients with coexisting LPRD and ETD following PPI therapy, but the evidence remains inconclusive [11]. Further research is required to determine whether acid suppression alone is sufficient to alleviate ETD symptoms or if additional interventions are necessary.

Despite growing recognition of the potential link between LPRD and ETD, a limited number of studies have specifically evaluated the effect of PPI therapy on ETD symptoms. Understanding this relationship could help refine treatment strategies for patients with refractory ETD symptoms. This study aims to bridge this gap by assessing the impact of PPI therapy on ETD symptoms, and their potential correlation, via validated clinical scores that can be used in the simplest of clinical settings.

The primary objective of this study, conducted in a cohort of patients in Pakistan, was to evaluate the impact of PPI therapy on symptom severity in patients with ETD and coexisting LPRD. It leverages standardized diagnostic tools like ETDQ-7, RSI, and the Reflux Finding Score (RFS) to quantify disease burden and treatment outcomes. By focusing on the therapeutic impact of PPIs on ETD symptoms, it aims to offer evidence-based recommendations for managing this clinically significant yet under-researched interplay. By identifying affordable, scalable treatment approaches, the findings of this study will have implications for improving patient care in environments where both diagnostic and treatment resources are constrained.

## Materials and methods

This prospective observational study was conducted at the Benazir Bhutto Hospital, a tertiary care hospital in Rawalpindi, Pakistan, over 12 months (August 2023 to July 2024). A total of 126 adult patients presenting with signs and symptoms suggestive of both ETD and LPRD were recruited using non-probability consecutive sampling. Inclusion criteria were age between 18-60 years, clinical diagnosis of LPRD based on the RSI and RFS, and symptoms of ETD with an ETDQ-7 score ≥14.5 [9,10]. Exclusion criteria included confounders, such as prior ENT surgery affecting the Eustachian tube, acute infections or chronic diseases of the ear or elsewhere, symptoms unrelated to LPRD, smokers, patients with a history of allergies, and patients who did not adhere to follow-up visits or complete the prescribed treatment course. Ethical approval for the study was obtained from the ethical review board of Rawalpindi Medical University, Rawalpindi (reference no: ENT-25-49-2023), and the study was performed as per the ethical standards laid down in the 1964 Declaration of Helsinki. Written informed consent was obtained from all participants before data collection and publication, and they were assured of the anonymization of their data. Validated questionnaires, including the ETDQ-7, RSI, and RFS, were administered at baseline and after eight weeks of therapy to assess symptom severity and treatment response (Appendix A). Details of the tools used were as follows:

1. ETDQ-7: A validated tool for assessing ETD symptoms, scored on a scale of 1 (no problem) to 7 (severe problem), with a cutoff of ≥14.5 indicating clinically significant ETD [9].

2. RSI: A validated nine-item questionnaire assessing symptoms of reflux, with a total score >13 considered indicative of LPRD [10].

3. RFS: A scoring system used during laryngoscopic evaluation to quantify the severity of reflux-induced changes, with a total score >7 considered diagnostic for LPRD [10].

All participants received PPI therapy (40 mg omeprazole twice daily) for eight weeks, with instructions to adhere to standard reflux management protocols. These included dietary modifications i.e. restriction of spicy or oily foods or caffeine intake, and lifestyle changes i.e. head elevation while sleeping, no heavy meals three hours before sleeping, and a 10-minute walk after dinner. Baseline data were recorded, including demographics, clinical history, ETDQ-7, RSI, and RFS scores. Follow-up evaluations were conducted at four and eight weeks, during which the same questionnaires were administered. Data were collected and anonymized for analysis. Statistical analysis was performed using IBM SPSS Statistics for Windows, Version 26 (Released 2018; IBM Corp., Armonk, New York, United States). Continuous variables were expressed as mean±standard deviation (SD). Paired sample t-tests compared pre- and post-treatment scores for ETDQ-7, RSI, and RFS. The Pearson correlation coefficient assessed the relationship between ETDQ-7 scores and RSI/RFS scores both pre- and post-intervention. A p-value of <0.05 was considered statistically significant.

## Results

Out of the 126 patients who enrolled initially, 16 (12.7%) were lost to follow-up, leaving a final cohort of 110 participants. The majority of participants were female (70%), which may reflect gender differences in symptom reporting or disease prevalence. At baseline, all participants had clinically significant ETD (ETDQ-7 ≥14.5; mean score: 16.2 ± 1.8). LPRD was also confirmed, with mean RSI and RFS scores of 22.56 ± 6.23 and 11.42 ± 3.76, respectively (Table 1).

**Table 1:** Baseline characteristics of the study participants, including demographic and clinical variables. Data are presented as mean ± standard deviation (SD) or frequency (percentage), as appropriate. BMI: Body Mass Index; ETDQ-7: Eustachian Tube Dysfunction Questionnaire; RSI: Reflux Symptom Index; RFS: Reflux Finding Score

Characteristic	Mean ± SD or n (%)
Total participants (N)	110
Age (years)	34.2 ± 10.24
Gender	Male: 33 (30%) Female: 77 (70%)
BMI (kg/m²)	29.24±4.32
Baseline ETDQ-7 Score	16.2±1.8
Baseline RSI Score	22.56±6.23
Baseline RFS Score	11.42±3.76

An independent t-test revealed no significant differences in pre-intervention ETDQ-7, RSI, or RFS scores based on gender. Additionally, Pearson correlation tests indicated no significant association between age or BMI and symptom severity (p>0.05 for all comparisons). See Table 2 for details.

**Table 2:** Statistical analysis of demographic variables and outcome scores Independent t-tests were used to compare gender differences in ETDQ-7, RSI, and RFS scores, while Pearson’s correlation was used for age and BMI associations. BMI: Body Mass Index; ETDQ-7: Eustachian Tube Dysfunction Questionnaire; RSI: Reflux Symptom Index; RFS: Reflux Finding Score

Variable	Score	Mean ± SD	t-value	p-value
Gender	ETDQ-7	16.1±1.9 (Male), 16.2±1.8 (Female)	0.3492	0.7279
	RSI	22.3±5.8 (Male), 22.6±6.3 (Female)	0.5943	0.5542
	RFS	11.0±3.5 (Male), 11.5±3.8 (Female)	1.4467	0.1524
Age	ETDQ-7	16.2±1.8	0.0535	0.5787
	RSI	22.56±6.23	-0.0548	0.5693
	RFS	11.42±3.76	-0.1065	0.2682
BMI	ETDQ-7	16.2±1.8	-0.0539	0.5761
	RSI	22.56±6.23	-0.1309	0.1730
	RFS	11.42±3.76	0.1444	0.1322

**Figure 1:**
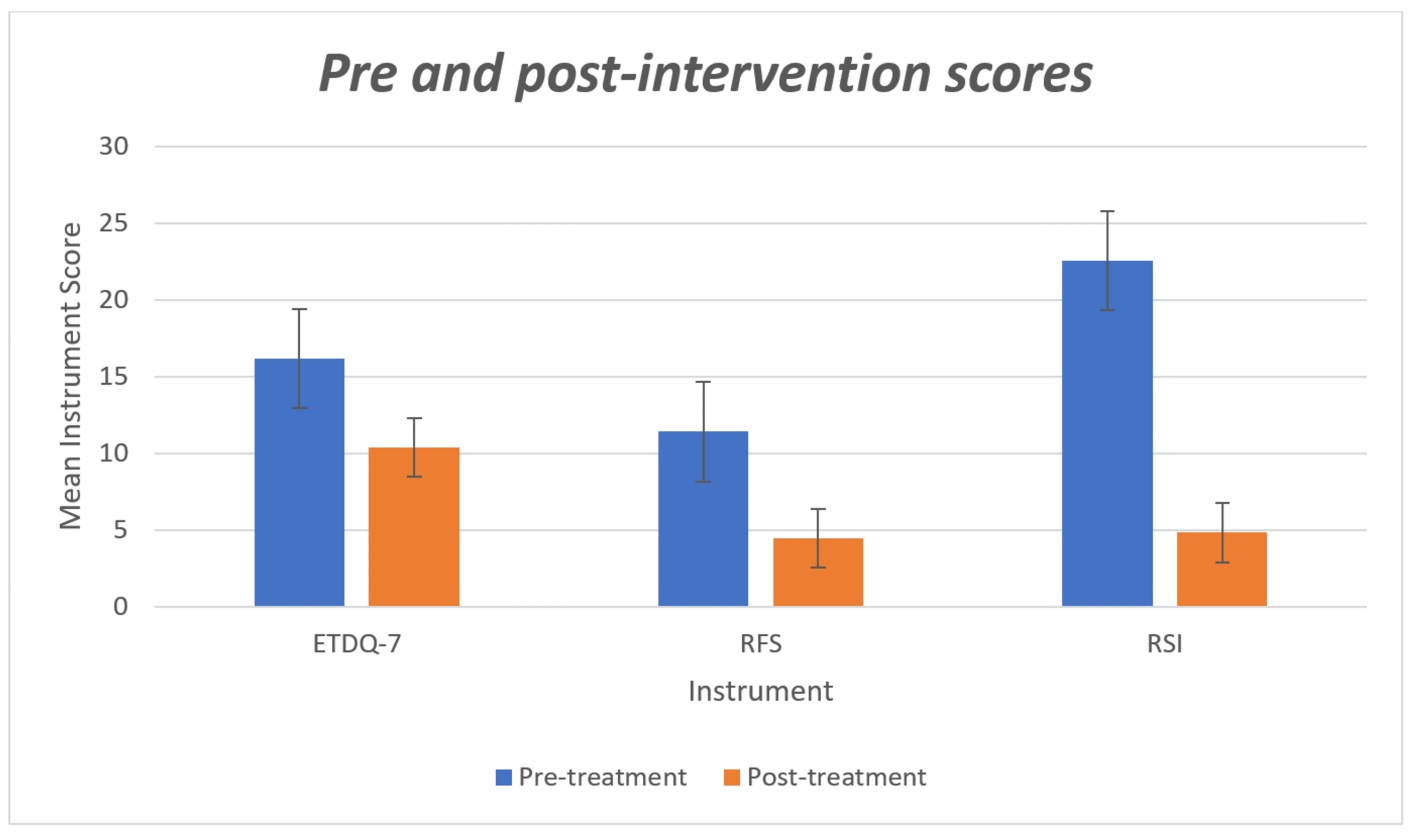
Pre and post-intervention ETDQ-7, RFS, and RSI scores ETDQ-7: Eustachian Tube Dysfunction Questionnaire; RSI: Reflux Symptom Index; RFS: Reflux Finding Score

Eight weeks of PPI therapy revealed a significant reduction in symptom scores following treatment. The mean ETDQ-7, RFS, and RSI scores showed a significant decrease after treatment (Figure 1).

These findings suggest that the intervention was effective in improving symptoms as measured by all three scales (Table 3).

**Table 3:** Pre- and post-treatment ETDQ-7, RFS, and RSI scores Paired t-tests were conducted for pre- vs. post-treatment comparisons. Effect size (r-value): Small: r<0.3; Medium: 0.3≤ r <0.5; Large: r≥0.5 SD: Standard deviation; ETDQ-7: Eustachian Tube Dysfunction Questionnaire; RSI: Reflux Symptom Index; RFS: Reflux Finding Score.

Variable	Pre-treatment (Mean±SD)	Post-treatment (Mean±SD)	t-value	p-value	Effect size (r-value)
ETDQ-7	16.20±10.47	10.40±5.47	6.32	<0.001	0.518
RFS	11.42±7.65	4.50±2.65	2.51	0.014	0.233
RSI	22.56±10.14	4.84±2.45	3.44	<0.001	0.313

A moderate positive correlation was observed between ETDQ-7 and RSI/RFS scores before and after PPI therapy. All the pre- and post-treatment correlations were highly significant (Table 4).

**Table 4:** Pearson correlation coefficients (r) and p-values for the relationship between ETDQ-7 and RSI/RFS scores before and after PPI therapy. ETDQ-7: Eustachian Tube Dysfunction Questionnaire; RSI: Reflux Symptom Index; RFS: Reflux Finding Score; PPI: Proton Pump Inhibitors

Comparison	Mean ± SD	r	p-value
Pre-treatment ETDQ-7 & RSI	(16.20±10.47) & (22.56±10.14)	0.391	p<0.001
Pre-treatment ETDQ-7 & RFS	(16.20±10.47) & (11.42±7.65)	0.437	p<0.001
Post-treatment ETDQ-7 & RSI	(10.40±5.47) & (4.84±2.45)	0.346	p<0.001
Post-treatment ETDQ-7 & RFS	(10.40±5.47) & (4.50±2.65)	0.515	p<0.001

These findings suggest a consistent relationship between the severity of ETD symptoms and reflux-related scores, further supporting the hypothesis that LPRD may contribute to the pathophysiology of ETD.

## Discussion

This study demonstrates a significant reduction in ETDQ-7, RSI, and RFS scores following an eight-week course of PPI therapy. The observed improvements highlight the potential interplay between LPRD and ETD, suggesting that addressing the reflux component may alleviate associated otologic symptoms. Importantly, this study provides new insights within a resource-constrained setting, where comorbidities often remain underdiagnosed and untreated.

Our findings are consistent with evidence indicating a link between LPRD and ETD. Lechien et al. reviewed how refluxate exposure damages the nasopharyngeal mucosa, impairing the Eustachian tube's pressure regulation and ventilation mechanisms [4]. This aligns with the reduction in ETDQ-7 scores observed in our cohort. Similarly, studies by Bhargava et al. and Jung et al. have reported improvements in otologic symptoms following reflux treatment, supporting the notion of shared pathophysiological pathways [7,10]. Our analysis demonstrated that demographic factors such as age, gender, and BMI did not significantly influence ETDQ-7, RSI, or RFS scores (Table 2). These findings suggest that symptom severity and response to PPI therapy are primarily driven by the underlying pathophysiology of LPRD and ETD rather than patient demographics. This supports the generalizability of our findings across different patient subgroups.

However, controversies remain regarding the effectiveness of PPIs in managing extra-esophageal manifestations of GERD. Hom et al. critically evaluated PPI efficacy, noting mixed results for symptoms like chronic cough and LPRD [12]. Aural fullness, on the other hand, is a shared symptom of both ETD and LPRD, with PPIs reported to offer symptomatic relief in some cases [12]. While our findings indicate a beneficial role of PPIs in ETD, the moderate correlations between RSI/RFS and ETDQ-7 scores suggest that additional factors, such as structural anomalies or allergic etiologies, may contribute to ETD symptomatology.

The pathophysiological link between LPRD and ETD is multifactorial. As Liu et al. elaborated, refluxate containing acid and pepsin disrupts the mucosal barrier, triggering inflammation and compromising the Eustachian tube's functionality [5]. Chronic exposure to such irritants impairs the tube's clearance mechanisms and may lead to epithelial remodeling and persistent dysfunction. As Lechien et al. recently highlighted, the role of the refluxate in triggering inflammatory cascades within the upper aerodigestive tract extends to alterations in mucosal immunity and epithelial integrity [13]. Zhen et al. identified a significant inverse correlation between RSI scores and Eustachian tube patency in patients with otitis media with effusion (OME). Their findings suggest that LPRD may contribute to ETD through impaired ciliary clearance and mucosal edema. This aligns with the observed relationship between RSI and ETDQ-7 scores in the current study, further substantiating the role of reflux in otologic conditions [14]. These processes provide further evidence for the multifactorial nature of LPRD-associated ETD, reinforcing the plausibility of a direct mechanistic link. These mechanisms explain the observed improvements in our study after PPI therapy, which likely mitigates refluxate-induced damage.

The implications of these findings are particularly significant for resource-limited settings. Given the high prevalence of untreated reflux disorders and the limited availability of advanced diagnostic modalities, the use of validated tools such as the ETDQ-7 and RSI provides a practical approach for diagnosis and monitoring. Moreover, PPIs represent a cost-effective intervention that can be readily implemented in such environments. The findings of Zhen et al. further highlight the potential role of RSI as a predictive tool for identifying patients who may benefit from anti-reflux therapies [14]. Incorporating RSI in routine evaluations could optimize treatment strategies, especially in patients with concurrent ETD and elevated reflux symptoms.

This study has several strengths, including its prospective design, use of validated questionnaires, and focus on a clinically significant yet under-researched comorbidity. However, there are limitations. The single-center nature of the study and reliance on subjective symptom scores may limit generalizability. The non-random consecutive sampling and lack of a control group introduce potential bias. These limitations could be addressed in future randomized controlled trials incorporating blinding. Additionally, the eight-week follow-up period precludes the assessment of long-term outcomes.

Future research should aim to validate the observed associations in diverse populations and explore alternative therapeutic approaches to build on these findings. Alginate-based formulations, for example, may offer additional benefits by forming a protective barrier against refluxate. Future research should further explore the applicability of tubomanometry (TMM) as utilized by Zhen et al. for objectively assessing Eustachian tube function in patients with LPRD [14]. This could provide a standardized framework to evaluate the impact of anti-reflux therapies on ETD across diverse patient populations. Longitudinal studies assessing relapse rates, adherence challenges, and the interplay of other factors, such as dietary habits and allergies, are also warranted. Finally, mechanistic investigations using advanced imaging or molecular techniques could further elucidate the pathophysiology linking LPRD and ETD.

## Conclusions

This study provides some evidence for the significant role of LPRD in the pathophysiology of ETD. Through an eight-week course of PPI therapy, we observed a notable improvement in ETDQ-7, RSI, and RFS scores, suggesting that PPI treatment effectively alleviates both the symptoms of LPRD and its associated otologic manifestations. The moderate correlations between reflux-related scores and ETD symptom severity further reinforce the link between these conditions. Additionally, demographic factors such as age, gender, and BMI did not significantly influence symptom severity, indicating that the pathophysiology of LPRD and its impact on ETD are not driven by these variables.

Our findings highlight the potential of PPIs as a cost-effective therapeutic option, particularly in resource-limited settings, given their observed efficacy and the frequent underdiagnosis of reflux disorders in such contexts. Despite the promising results, future studies with larger, more diverse populations and randomized controlled trials are needed to validate these associations and evaluate the long-term efficacy of PPI therapy in managing LPRD-associated ETD. Investigating complementary treatment strategies such as alginate-based formulations and objective measures like tubomanometry, will help refine therapeutic approaches and provide deeper insights into the underlying mechanisms.

In conclusion, this study contributes to our understanding of the relationship between LPRD and ETD, offering a promising avenue for the management of these overlapping conditions. By addressing reflux as a potential contributor to ETD, clinicians may improve patient outcomes and enhance the quality of care for those suffering from these often underappreciated comorbidities.

## Appendices

Appendix A: Validated questionnaires and clinical assessment sheet

Participant Demographic and Clinical Information

Patient ID: _____________________________

Age: __________ Sex: ☐ Male ☐ Female ☐ Other

Occupation: _____________________________

Duration of symptoms (months): __________

History of smoking: ☐ Yes ☐ No

Allergic rhinitis history: ☐ Yes ☐ No

History of gastroesophageal reflux disease (GERD): ☐ Yes ☐ No

Use of PPI (proton pump inhibitors): ☐ Yes ☐ No

ENT examination findings (summary): _____________________________

Laryngoscopy performed: ☐ Yes ☐ No

Other relevant findings or comments: _____________________________

Eustachian Tube Dysfunction Questionnaire (ETDQ-7)

Please rate each item from 1 (No problem) to 7 (Severe problem)

**Table 5:** Eustachian Tube Dysfunction Questionnaire (ETDQ-7) [9]

Item	Question	1	2	3	4	5	6	7
1	Pressure or clogged feeling in your ears?	☐	☐	☐	☐	☐	☐	☐
2	Pain in your ears?	☐	☐	☐	☐	☐	☐	☐
3	A feeling that your ears are clogged or "under water"?	☐	☐	☐	☐	☐	☐	☐
4	Crackling or popping sounds in your ears?	☐	☐	☐	☐	☐	☐	☐
5	Ringing in your ears?	☐	☐	☐	☐	☐	☐	☐
6	Feeling that your hearing is muffled?	☐	☐	☐	☐	☐	☐	☐
7	Feeling that your ears are not clear after yawning or swallowing?	☐	☐	☐	☐	☐	☐	☐

Reflux Symptom Index (RSI)

Rate each symptom from 0 (No problem) to 5 (Severe problem)

**Table 6:** Reflux Symptom Index (RSI) [10]

Item	Question	0	1	2	3	4	5
1	Hoarseness or a problem with your voice	☐	☐	☐	☐	☐	☐
2	Clearing your throat	☐	☐	☐	☐	☐	☐
3	Excess throat mucus or postnasal drip	☐	☐	☐	☐	☐	☐
4	Difficulty swallowing food, liquids, or pills	☐	☐	☐	☐	☐	☐
5	Coughing after eating or after lying down	☐	☐	☐	☐	☐	☐
6	Breathing difficulties or choking episodes	☐	☐	☐	☐	☐	☐
7	Troublesome or annoying cough	☐	☐	☐	☐	☐	☐
8	Sensation of something sticking in your throat or a lump in your throat	☐	☐	☐	☐	☐	☐
9	Heartburn, chest pain, indigestion, or stomach acid coming up	☐	☐	☐	☐	☐	☐

Reflux Finding Score (RFS)

Assessed by a clinician during laryngoscopy

**Table 7:** Reflux Finding Score (RFS) [10]

Item	Finding	Score range
1	Subglottic edema (pseudosulcus)	0–2
2	Ventricular obliteration	0–2
3	Erythema/hyperemia	0–4
4	Vocal fold edema	0–4
5	Diffuse laryngeal edema	0–4
6	Posterior commissure hypertrophy	0–4
7	Granuloma/granulation tissue	0–3
8	Thick endolaryngeal mucus	0–2
